# Reduced cholinergic tone and hippocampal‐dependent cognitive impairments in APP^NL‐F^ mice

**DOI:** 10.1002/alz70855_107535

**Published:** 2025-12-24

**Authors:** Alycia M Crooks, Kate M. Onuska, Taylor W Schmitz, Lisa M Saksida, Timothy J Bussey, Vania F Prado, Marco AM Prado

**Affiliations:** ^1^ Lawson Health Research Institute, London, ON, Canada; ^2^ Robarts Research Institute, London, ON, Canada; ^3^ University of Western Ontario, London, ON, Canada; ^4^ Schulich School of Medicine and Dentistry, London, ON, Canada

## Abstract

**Background:**

The prevailing model of Alzheimer's disease (AD) proposes that the accumulation of misfolded amyloid‐beta (Aβ) peptides drives tau protein hyperphosphorylation and neuroinflammation, leading to neurodegeneration and cognitive decline. However, current therapeutic approaches targeting Aβ show only subtle effects on cognitive deficits. Interestingly, the loss of cholinergic neurons of the basal forebrain precedes and predicts pathology and neurodegeneration in AD. The vesicular acetylcholine transporter (VAChT), a key regulator of cholinergic signalling, functions at the presynaptic terminal by loading acetylcholine (ACh) into vesicles for secretion. Notably, VAChT expression is significantly reduced in AD brains, with changes observed early in disease progression. Whether these early changes in cholinergic synaptic function contribute to hippocampal dysfunction remains unclear. Using the APP^NL‐F^ mouse model of AD, which expresses humanized Aβ with familial AD mutations, this study investigates the interplay between cholinergic signalling and hippocampal‐dependent cognitive function.

**Method:**

ACh signalling was recorded using fibre photometry and the GRAB_ACh_ sensor in the hippocampal CA1 region of freely behaving APP^NL‐F^ and control mice performing the Trial‐Unique delayed Nonmatching‐to‐Location (TUNL) task. This task assesses spatial working memory, a cognitive domain impaired in AD. Large and small separation windows were used to challenge spatial discrimination.

**Result:**

Analysis of VAChT levels have shown some dynamic regulation, but with aging VAChT levels decreased substantially. Recordings of ACh dynamics in the hippocampal CA1 region revealed behaviour‐associated changes, particularly during screen approaches, touches, and predominantly reward responses. Cholinergic tone decreases substantially in response to reward, demonstrating tonic ACh signalling that can change in response to specific stimuli. Young APP^NL‐F^ mice, tested before substantial plaque accumulation, exhibit deficits in spatial working memory during challenging conditions, which associated with reduced cholinergic responses. The deficit in cholinergic tone in APP^NL‐F^ mice closely mirrors the cholinergic dysfunction observed in the forebrain VAChT knock‐out mice, which showed pronounced impairments in learning, memory, and ACh signalling during the TUNL task.

**Conclusion:**

These findings highlight the role of early deficits in cholinergic synaptic function associated with spatial memory impairments before overt pathology in APP^NL‐F^ mice. Future experiments aim to develop strategies to rescue memory and cholinergic tone dysfunction.

